# A mechanistic understanding of the relationship between skin innervation and chemotherapy-induced neuropathic pain

**DOI:** 10.3389/fpain.2022.1066069

**Published:** 2022-12-13

**Authors:** Cristina Meregalli, Laura Monza, Joost L. M. Jongen

**Affiliations:** ^1^ School of Medicine and Surgery, Experimental Neurology Unit and Milan Center for Neuroscience, University of Milano-Bicocca, Monza, Italy; ^2^ Department of Neurology, Brain Tumor Center, Erasmus MC Cancer Institute, Rotterdam, Netherlands

**Keywords:** cutaneous sensory endings, IENF density, chemotherapy-induced peripheral neuropathy, neuropathic pain, skin biopsy

## Abstract

Neuropathic pain is a frequent complication of chemotherapy-induced peripheral neurotoxicity (CIPN). Chemotherapy-induced peripheral neuropathies may serve as a model to study mechanisms of neuropathic pain, since several other common causes of peripheral neuropathy like painful diabetic neuropathy may be due to both neuropathic and non-neuropathic pain mechanisms like ischemia and inflammation. Experimental studies are ideally suited to study changes in morphology, phenotype and electrophysiologic characteristics of primary afferent neurons that are affected by chemotherapy and to correlate these changes to behaviors reflective of evoked pain, mainly hyperalgesia and allodynia. However, hyperalgesia and allodynia may only represent one aspect of human pain, i.e., the sensory-discriminative component, while patients with CIPN often describe their pain using words like annoying, tiring and dreadful, which are affective-emotional descriptors that cannot be tested in experimental animals. To understand why some patients with CIPN develop neuropathic pain and others not, and which are the components of neuropathic pain that they are experiencing, experimental and clinical pain research should be combined. Emerging evidence suggests that changes in subsets of primary afferent nerve fibers may contribute to specific aspects of neuropathic pain in both preclinical models and in patients with CIPN. In addition, the role of cutaneous neuroimmune interactions is considered. Since obtaining dorsal root ganglia and peripheral nerves in patients is problematic, analyses performed on skin biopsies from preclinical models as well as patients provide an opportunity to study changes in primary afferent nerve fibers and to associate these changes to human pain. In addition, other biomarkers of small fiber damage in CIPN, like corneal confocal microscope and quantitative sensory testing, may be considered.

## Introduction

Neuropathic pain is defined as “pain that arises as a direct consequence of a lesion or disease affecting the somatosensory system” (https://www.iasp-pain.org/advocacy/global-year/neuropathic-pain/) (1). It is recognized as the most common neurologic complication in patients treated with cisplatin, oxaliplatin, taxanes like paclitaxel, vinca-alkaloids, proteasome inhibitors like bortezomib and immune-modulatory drugs like thalidomide (2–4). The prevailing hypothesis is that axonal swellings, containing accumulations of mitochondria, usually occur early in the course of distal symmetric peripheral neuropathies, while (epi)dermal nerve fiber loss and degenerative Schwann cell changes occur as late consequences (5–7). Kroigard and colleagues (8–10) have described in detail clinical characteristics and the results from nerve conduction studies (NCS), quantitative sensory testing (QST) and IENFD measurements in patients with oxaliplatin or docetaxel-induced peripheral neuropathy. IENFD measurements using PGP9.5 immunohistochemistry provide a robust, objective and minimally invasive way to quantify epidermal innervation. It does, however, suffer from suboptimal sensitivity and lack of scalability/labor intensiveness, although the latter can potentially be overcome in the future using automated deep learning algorithms.

QST is a method to detect sensory deficits and evoked neuropathic pain, which usually includes mechanical, thermal, or pressure pain threshold. A reduction in different nerve fiber populations is related to a deficit of specific nerve fibre sensitivities. In particular, Abeta-fibre loss is associated with loss of vibration perception, light touch sensation and elevated mechanical detection thresholds. Heat detection and heat pain thresholds provide valuable information about C-fibre loss. Finally, Adelta-fibre loss is linked to decreased pinprick stimuli, mechanical pain and cold detection (11).

Apart from intraepidermal nerve fiber density and QST, there are other biomarkers of chemotherapy-induced peripheral neuropathy (CIPN), like neurofilament light chain for axonal damage (12–14) and corneal confocal microscopy (CCM). The latter is a non-invasive ophthalmologic imaging technique which quantifies small nerve fibre abnormalities in the sub-basal nerve plexus of the cornea (11).

Diagnostic sensitivity and several associations between the severity of intraepidermal nerve fiber loss, severity of NCS or QST abnormalities in oxaliplatin-treated patients vs. neurpathic pain were studied (8–10). Regarding CCM, different studies have shown corneal nerve microstructure and corneal sensitivity changes in patients receiving oxaliplatin (15). In addition, imaging of the corneal sub basal nerve plexus was reported as biomarker for nerve regeneration in CIPN (16). On the other hand, in oxaliplatin- and docetaxel-induced polyneuropathy changes in QST, but not in CCM, were reported (17). Neuropathic pain may arise as a consequence of loss and/or degeneration of nerve fibers, from hyperexcitability of spared and sensitized nociceptive afferents as well as from reinnervation (18).

### Innervation of the epidermis and neuropathic pain

**Figure 1:**
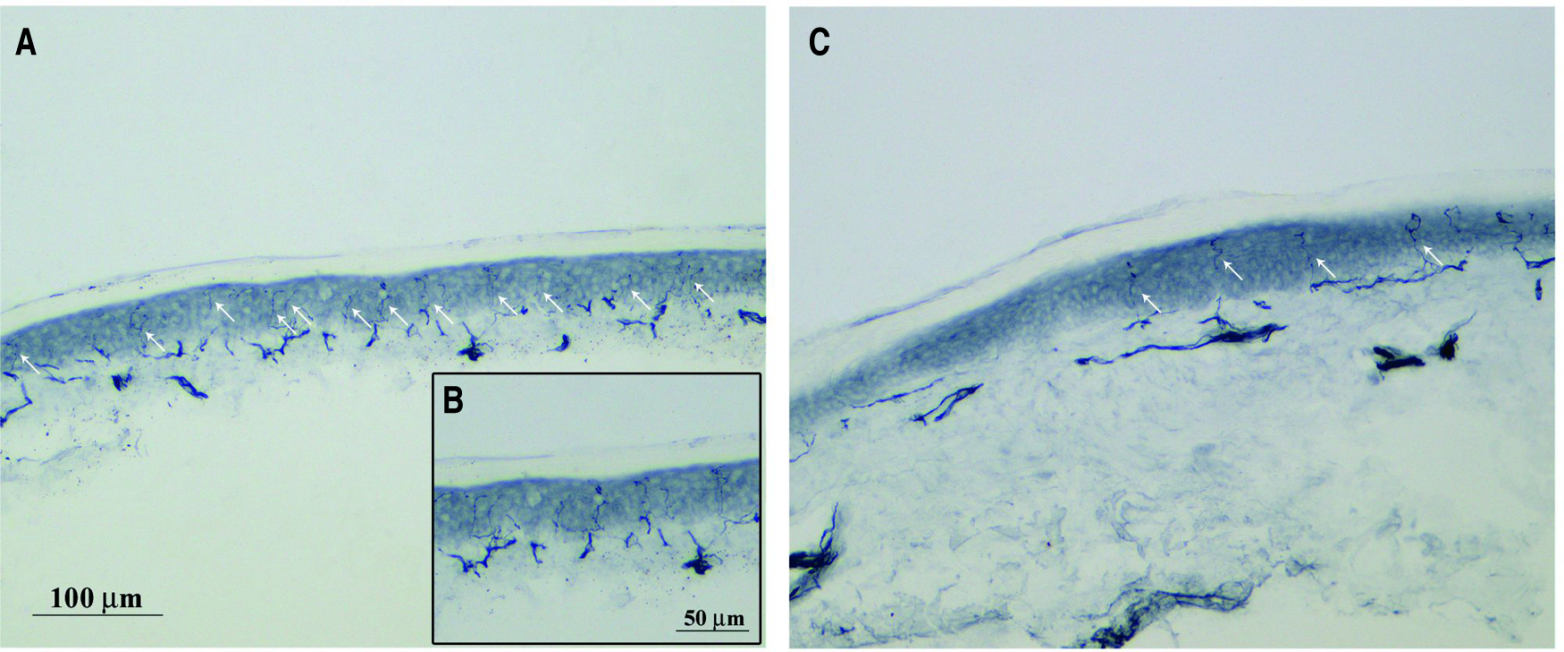
PGP 9.5 immunohistochemical staining free intraepidermal nerve fibers endings. Representative images of skin biopsy from a control (**A**), higher magnification in (**B**) and PTX-treated rats (**C**) after 4 weeks of treatment (bar A and C = 100 μm, bar B = 50 μm). PGP9.5 immunohistochemical staining was performed to measure the intraepidermal nerve fibers (IENF) density using a previously published protocol (26). Briefly, glabrous skin from the plantar hindpaw were fixed, cryoprotected and serially cut in 20 μm-thick sections. Sections were immunostained with rabbit polyclonal anti-protein gene product 9.5 (PGP 9.5; ProteinTech, Manchester, United Kingdom) using a free-floating protocol. The total number of PGP 9.5-positive IENF crossing the dermal-epidermal junction was counted under a light microscope at 40× magnification and then the IENF density was expressed as number IENF/length of epidermis (mm).

In CIPN nor in other neuropathic pain conditions few studies have confirmed positive or negative associations between cutaneous innervation and the severity of neuropathic pain (19, 20), while many described no consistent correlations (21–24). IENF density and onset of neuropathic pain behaviors is not consistent across neuropathic pain models. One reason may be mixed pathology, for example in painful diabetic neuropathy where ischemic pain may be superimposed on purely neuropathic pain. Another reason may be the fact that selective degeneration of subsets of intraepidermal nociceptive fibers (IENF), which cannot be detected using the pan axonal marker PGP9.5, may drive hypersensitivity and has been linked to neuropathic pain. Although PGP9.5 labeling can be obtained by simple indirect immunofluorescence and bright-field immunohistochemistry on free-floating sections for IENF density quantification (25), it is important to remember that using immunolabeling of PGP 9.5 it is possible to detect intact free intraepidermal nerve fibers endings (see Figure 1), but without any distinction in various subtypes of sensory nerve fibers (27). It is known that the primary afferent sensory fibers in the epidermis and dermal layers of the skin can be structurally defined as Aβ, A∂ and C-fibers (28). In addition to this morphological classification, neurofilament 200 (NF200) is a typical immunohistochemical marker of myelinated fibers (29). C-fibers can be further subdivided using immunohistochemical/molecular markers such as calcitonin gene related peptide (CGRP), substance *P*, ion channels, transient receptor potential (TRP) family receptors (like the vanilloid receptor 1 TRPV1) and endocannabinoid receptors (like CB1) (30). These markers define the subclass of peptidergic nociceptors (named so, because they contain neuropeptides). In contrast, non-peptidergic nociceptors are C-fibers with free endings in the epidermis which are often identified by the binding isolectin B4 (IB4) or through expression of the purinergic receptor P2X_3_ (28–31). These subgroups of C-fibers may convey differential susceptibility to toxic injury, possibly associated with neuropathic pain. Peptidergic and nonpeptidergic nerve fibers have different innervation patterns in the epidermis and dermis (32), in particular there is a much denser innervation of the epidermis by P2X_3_-IR fibers in comparison with the peptidergic or CGRP positive ones (33, 34).

### Human pain: the lateral and the medial pain system

**Figure 2:**
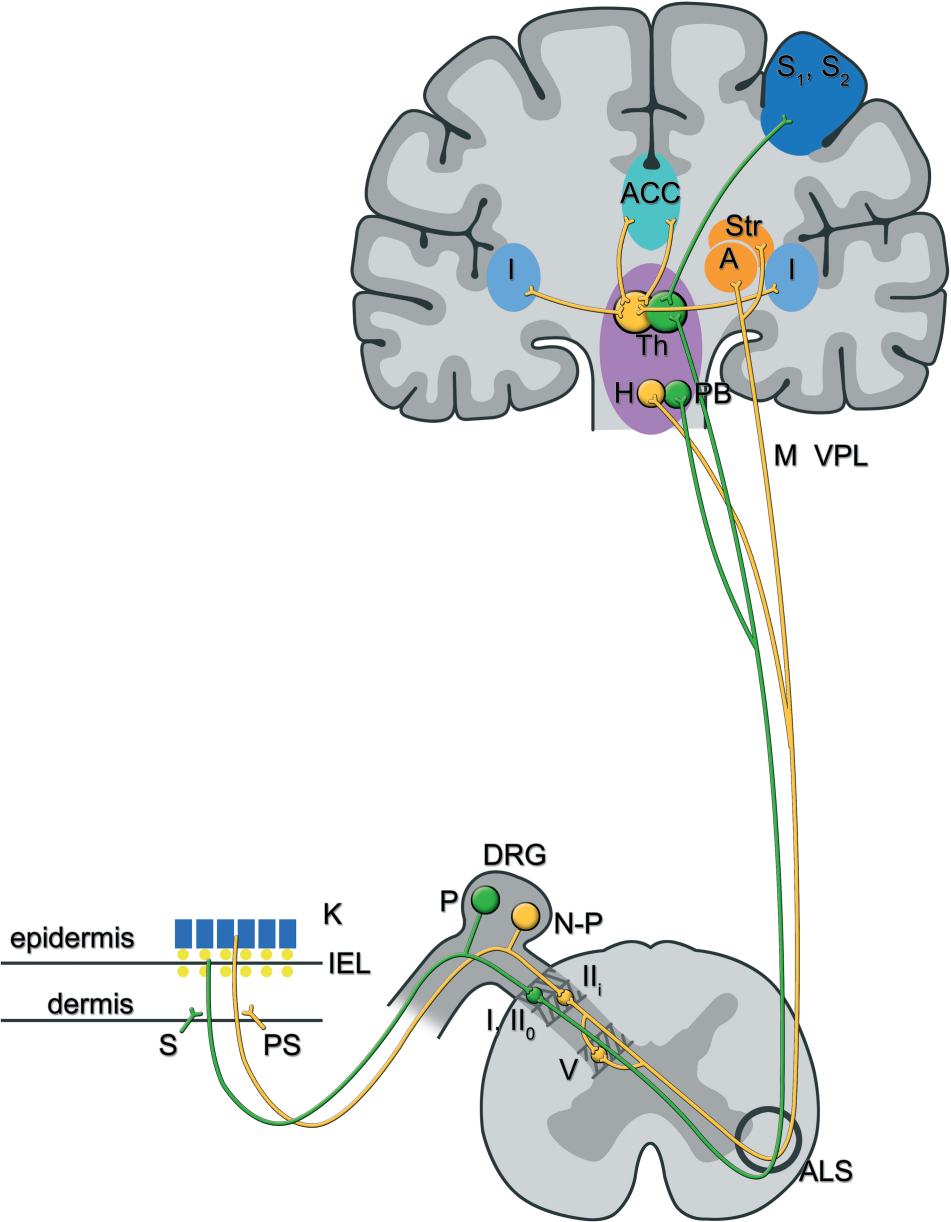
The lateral and medial pain ascending pathways in chemotherapy-induced peripheral neurotoxicity: afferents of peptidergic and non-peptidergic C-fibres that form free nerve endings in the skin terminate in the superficial lamina of the spinal dorsal horn. In particular, peptidergic C-fibres synapse on lamina I and lamina II_o_ neurons, whereas non-peptidergic C-fibres synapse on lamina II_i_ (inter)neurons (39). Those second order pain neurons ascend, directly (lamina I and II_o_ neurons) (38) or indirectly (lamina II_i_ neurons, mostly *via* neurons in lamina V) (36), through the anterolateral system and project to brainstem and forebrain areas, amongst others the parabrachial nuclei and thalamus for peptidergic input, versus the amygdala, hypothalamus and striatal nuclei for non-peptidergic input. Functional MRI and PET studies have shown that separate brain areas process distinctive pain components: sensory-discriminative aspects of pain are processed in the contralateral primary and secondary sensory cortex, while affective components are processed in the hypothalamus, the contralateral amygdala and bilateral anterior cingulate cortex and insula (40, 41, 45). It is suggested that the pathway originating from peptidergic primary afferents (also called the lateral pain system-in green) contributes to the sensory-discriminative aspect of pain, while the pathway originating from non-peptidergic primary afferents (also called the medial pain system-in yellow) contributes to the affective component of the pain experience (34, 38, 39). In line with the clinical observation that patients with CIPN more often complain about unpleasant than about intense pain sensations, the non-peptidergic or lateral pain system may predominate in those patients (27, 32, 42). Abbreviations: K, keratinocytes; S, sympathetic; PS, parasympathetic; DRG, dorsal root ganglion; P, peptidergic sensory neuron; N-P, non peptidergic sensory neuron; I, lamina I of the dorsal horn; II_o_, outer part of lamina II of the dorsal horn; II_i_,  inner part of lamina II of the dorsal horn; V, lamina V; ALS, anterolateral system; PB, parabrachial nuclei; Th, thalamus; H, hypothalamus; Str, striatal nuclei; S1, S2, somatosensory (association) cortex; A, amygdala; ACC, anterior gyrus cinguli; I, insula.

In contrast to experimental animal models of neuropathic pain, clinical pain research has the advantage to measure spontaneous pain instead of nociceptive reflexes (8, 9). Besides, humans have the ability to talk and thus differentiate between specific neuropathic pain components. Since the epidermis is predominantly innervated by non-peptidergic nociceptors (35) and since neuropathic pain is often qualified as both superficial as well as annoying, tring, dreadful etc., it was suggested that the predominant fibers in the most superficial layer of the skin (i.e., epidermal non-peptidergic nerve fibers) are correlated with the affective component of neuropathic pain (36). The McGill Pain questionnaire (37, 38) is a reliable and extensively validated test in many languages that was specifically designed to discern the sensory-discriminative, affective and evaluative components of neuropathic pain. Bechakra and colleagues recently published two papers, one in a rat-model of nerve-injury induced pain (34) and one in patients with bortezomib-induced peripheral neuropathy (BIPN) (39), in which they suggested that selective degeneration of non-peptidergic nerve fibers may directly or indirectly (*via* parasympathetic sprouting) (40), contribute to the affective and evaluative component of neuropathic pain in patients with BIPN. Impaired regeneration of peptidergic nerve fibers on the other hand may contribute to the sensory-discriminative component of neuropathic pain in BIPN patients (39). The situation may be different in more chronic neuropathies like (painful) diabetic neuropathy and chronic idiopathic axonal polyneuropathy, in which neuropathic pain intensity appears to be associated with increased (epidermal) sprouting of CGRP fibers (41, 42). It has been suggested that separate anatomical pathways exist for these respective components (26, 43), the so-called lateral and medial pain system (44). A schematic presentation of the medial and lateral and medial pain ascending pathways in chemotherapy-induced peripheral neurotoxicity is reported in Figure 2 (evidence is presented in the following citations: 34, 39, 43–50). Since non-peptidergic fibers are overrepresented in the epidermis, and since the degeneration of these fibers seems to be associated with the affective component of neuropathic pain, it is recommended to use a numerical rating scale (NRS) for pain unpleasantness in addition to an NRS for pain intensity for patients with CIPN-associated neuropathic pain.

## Mechanistic insight into CIPN related neuropathic pain

There is a wealth of clinical research on CIPN and of emerging pharmacological strategies for its management, which is reviewed in numerous citations (4, 50–51). However, our review focuses on the relationship between skin innervation and chemotherapy-induced neuropathic pain, which has been studied less extensively. Many studies report the loss of IENF density associated with diabetic neuropathy (52) and CIPN (53, 54), although the relationship with the development of neuropathic pain is unclear (55–58). Intraepidermal nerve terminals, arising from unmyelinated and thinly myelinated somatosensory fibers in the dermis, are exposed to inflammatory mediators which sensitize those peripheral sensory neurons (59) and are thus involved in the pathogenesis of neuropathic pain. The hyperexcitability of sensory neurons induced by the release of the neurotransmitters from their peripheral endings as well as alterations in ion-channel expression in those neurons is subsequently followed by central sensitization *via* spinal as well as supraspinal mechanisms, the latter involving ascending pathways (60). Together, peripheral and central sensitization mechanisms result in a neuropathic pain phenotype.

Since the pathogenic mechanisms underlying evoked neuropathic pain associated with CIPN development are still not fully understood, *in vivo* animal models may be useful not only to fill this knowledge gap but also to test promising pharmacological strategies aimed at preventing the development of neuropathy and/or neuropathic pain.

Several rodent models have evidenced that loss of nerve fibers innervating the skin is involved in the initiation and persistence of neuropathic pain, for example in paclitaxel- (56, 61), oxaliplatin- (55), vincristine (62), cisplatin- (63) and bortezomib-induced peripheral neuropathies (4, 64, 65). In the majority of clinical (66) and preclinical studies, skin biopsies (indicative of small nerve fibres damaged in the skin) are used to examine IENF density in the glabrous skin and evaluated by immunostaining using the pan-axonal marker PGP9.5 (67), whereas electrophysiological tests or conventional nerve histology can be performed to examine the pathology in large fiber neuropathies (68). A significant decline in IENF density appears prior to the onset of damage in more proximal nerves and sensory ganglia (69). The immunomodulatory agents minocycline and immunoglobulin, administered 24 h just before and during chemotherapy, were able to prevent mechanical hyperesthesia (exaggerated responses to noxious stimuli)/allodynia (responses to non-noxious stimuli) associated with a decrease in IENF density induced by paclitaxel and oxaliplatin (46, 47) and bortezomib (70).

### Nerve fiber degeneration and regeneration in CIPN and neuropathic pain

Patients suffering from CIPN-associated neuropathic pain experience both spontaneous as well as stimulus-dependent pain that manifests as hyperalgesia and allodynia (71). Pain-like behaviours in animal models of CIPN are mainly studied through tests based on stimulus-evoked responses to define the onset, severity and duration of mechanical allodynia and/or thermal hyperalgesia (72). As mentioned earlier, the association between pain-like behaviours and IENF density is not clear, i.e., some studies suggest that neuropathic pain behavior is associated with increased IENF density, while other studies suggest an inverse correlation. This may be related to re-innervation in epidermis and dermis of rats after nerve injury, selective degeneration of subsets of nociceptors (62, 73) and to the fact that specific subsets of nociceptors serve specific aspects of neuropathic pain (34).

Following a sciatic nerve lesion in rats, peptidergic nerve fibers demonstrated early and increased nerve sprouting from uninjured afferents resulting in a faster restoration of nociception and increased IENF density (74) as compared to non-peptidergic primary afferents, which took more time to recover than the peptidergic ones in a chronic constriction injury model (75). In addition, changes in the interactions between autonomic fiber types (i.e., sympathetic vs. parasympathetic) and sensory fibers occur in rat skin after peripheral nerve injury, suggesting involvement of autonomic nerve fibers in neuropathic pain mechanisms (76, 77). Regarding CIPN, it has been shown that paclitaxel induces the degeneration of CGRP and Substance P positive peptidergic nerve fiber terminals in the skin, leading to the onset of pain-like behaviors (73). However, other authors reported that vincristine induces a reduction of nonpeptidergic IENF density while it did not affect pepdidergic nerve fibers, resulting in the development of mechanical allodynia as a consequence of selective damage of vincristine to nonpeptidergic population (62). Thus, epidermal nerve fiber denervation and regeneration are probably not the only factors involved in the pathogenesis of neuropathic pain. Data from the literature suggest a possible key role of mitochondrial dysfunction and cutaneous neuroimmune interactions.

### Mitochondria dysfunction in CIPN-related neuropathic pain

In order to maintain epidermis integrity, IENFs are continuously subjected to a process of remodelling (78). This process implicates a high energy demand and may explain the high vulnerability of IENFs to the mitotoxic effects of antineoplastic agents (7). In the last few years, several preclinical studies have shown the effectiveness of pharmacological strategies aimed to improve mitochondrial abnormalities in reducing and or preventing CIPN and CIPN-associated neuropathic pain (79). This evidence led to the hypothesis that the mitochondrial dysfunction induced by antineoplastic agents is implicated in the onset and maintenance of CIPN (7). The prevailing idea is that chemotherapy can directly or indirectly cause damage to mitochondria that lead to a reduction in cellular bioenergetic capacity and an increased release of nitric oxide and superoxide. Nitro-oxidative stress and mitochondrial dysfunction can induce IENF degeneration and an increase in spontaneous discharges of damaged IENFs leading to neuropathic pain (79). For instance, Ma et al. described a cisplatin-induced peripheral neurotoxicity mouse model, in which they demonstrated that loss of IENF is correlated with bioenergetic deficits in peripheral nerves (80). In addition, Shim et al. reported cisplatin-induced mechanical hypersensitivity due to peripheral oxidative stress, sensitizing mechanical nociceptors (81). In line with this hypothesis, pharmacological agents such as pifithrin-µ (82, 83) that prevent p53 mitochondrial accumulation, as well as HDAC6 inhibitors (84) like metformin (85) and other compounds acting through reduction of nitro-oxidative stress (64), have shown to prevent and/or reduce IENF loss.

### The contribution of keratinocytes and immune cells to CIPN-associated neuropathic pain

Apart from associations of neuropathic pain intensity with nerve fiber degeneration or regeneration and mitochondrial dysfunction, a relationship with epidermal macrophages, Schwann cells and keratinocytes have been described in humans with neuropathic pain, although not specifically in patients with CIPN. Cutaneous cells (i.e., keratinocytes, the predominant epidermal cell type) and immune cells (i.e., macrophages, neutrophils, lymphocyte cells, mast cells and Langerhans cells) contribute to peripheral sensitization *via* their interactions with nociceptors (86).

#### Involvement of keratinocyte in CIPN

Keratinocytes express several receptors and ion channels like, for example, TRPV1–4 (87). Similarly, Langerhans cells (88) express, amongst others, T-type calcium channels, interleukin receptors, cannabinoid receptors, calcitonin receptor-like receptor (89–91). As an example of neuro-immune-cutaneous interactions, Cav3.2 T type calcium -channels are abundantly expressed within skin nerve endings thus regulating neuronal excitability and stimulus-evoked pain (92, 93).

Proposed mechanisms by which keratinocytes modulate neighboring neuronal and immune cells include the production and release of several neurotransmitters, chemokines (94), neuropeptides and/or cytokines, both *via* direct physical relationship between keratinocytes and cutaneous afferent fibers as well as *via* synaptic-like contacts (95) and/or contact with Schwann cells (96). In complex regional pain syndrome and post-herpetic neuralgia, increased activity and expression of voltage-gated sodium channels (Na_v_) on keratinocytes resulted in increased epidermal ATP release and excessive activation of P2X receptors on primary sensory axons (97). Moreover, it was reported that paclitaxel-induced keratinocyte damage and ectopic expression of matrix-metalloproteinase 13 (MMP-13) in the skin of a zebrafish model of CIPN is a fundamental event that precedes axonal degeneration (98). The inducers of this event could be mitochondrial damage and reactive oxygen species formation (99), as mentioned earlier. Not surprisingly, selective inhibition of MMP-13 improved skin defects and rescued paclitaxel-induced epithelial damage and neurotoxicity TRPV1 is expressed in a subpopulation of unmyelinated C and thinly myelinated A∂ nociceptors that mediate responsiveness to capsaicin and heat (100). Although little is known about the role of TRPV1 in keratinocytes, a recent study of Pang and colleagues reported that selective keratinocyte stimulation in a conditional TRPV1-knockout mice model, in which TRPV1 was exclusively expressed in keratinocytes, is sufficient to evoke acute nociceptive-related responses (84). Recently, a study described the distribution of cannabinoid receptors in the skin and peripheral nervous system. More specifically, CB1 receptors are expressed on nociceptive nerve endings and dorsal root ganglion neurons, whereas CB2 receptors are expressed on immune cells and keratinocytes (101). Endocannabinoids, such as 2-arachidonoylglycerol (2-AG) and N-arachidonoylethanolamine (AEA), are neurotransmitters derived from membrane phospholipid precursors. Their homeostasis is maintained by transporters and by the activity of fatty acid amide hydrolase (FAAH) and monoacylglycerol lipase (MAGL), enzymes that convert 2-AG to arachidonic acid and glycerol and that convert AEA to arachidonic acid and ethanolamine respectively (102). It has been described that chemotherapy induces increased expression of FAAH and MAGL in the footpads of experimental animals, which subsequently causes a decrease in 2-AG and AEA levels. As a consequence of decreased CB2 receptor activation, there is an increased sensitivity and response to nociceptor-stimulation leading to pain-like behaviors in animal models of CIPN 103). Studies from Ibrahim's laboratory tested the hypothesis that CB2 receptor activation stimulates the release of the endogenous opioid β-endorphin from keratinocytes, acting on primary afferent neurons *via* opioid receptors, thus reducing nociception (103).

#### Involvement of immune cells in CIPN

Recent evidence suggests that infiltrating immune cells can facilitate direct neuro-immune interactions (104). Interestingly, Shepherd et al. demonstrated that activation of the angiotensin II receptor on skin macrophages and consequent TRPA1 activation on sensory nerves is correlated with mechanical hypersensitivity and pain sensation (105). In addition, in the presence of IENF degeneration, activation of the skin's resident immune cells, i.e., Langerhans cells, was reported. Recently, murine models of paclitaxel and vincristine-induced painful neuropathies have shown a significant degeneration of IENF concomitant with the activation of PGP 9.5-positive Langerhans cells 106), suggesting a possible neuroimmune interaction mediated through an increased synthesis of proinflammatory cytokines by activated Langerhans cells (107), which are capable of sensitizing IENF terminals by evoking ectopic discharges and sensitizing C fibers (108). Finally, recent evidence has demonstrated that oxaliplatin induces changes in cutaneous mast cells that are correlated with the development of mechanical allodynia in mice (109). In particular, Sakamoto and colleagues reported a significant increase in total number and number of degranulated mast cells after oxaliplatin treatment, leading to the release of serine protease and activation of proteinase-activated receptor 2 (PAR2) in the skin, which caused mechanical allodynia. Similarly, release of tryptase from mast cells and activation of PAR2 in the skin lead to paclitaxel-induced mechanical allodynia through sensitization of TRPV1, TRPV4, TRPA1 receptors on primary afferent sensory neurons (110).

## Conclusion

In summary, degeneration and regeneration of unmyelinated C-fibers, mitochondrial dysfunction, keratinocytes, Langerhans cells and other cutaneous immune cells as well as TRP expressed by keratinocyte and sensory nerves, closely participate in sensory transduction and eventually contribute to the development of neuropathic pain in CIPN. In addition, selective epidermal nerve fiber degeneration and regeneration may affect two distinctive pain components, i.e., sensory-discriminatory vs. affective, *via* the lateral and medial pain systems respectively. Future studies should focus on the complex neuroimmuno interactions in the skin to define a strategy for the development of topical analgesics and neuroprotective drugs.
